# Supplementary material for: Strong coupling between a microwave photon and a singlet-triplet qubit
Source: Nat Commun. 2024 Feb 5;15:1068. doi: 10.1038/s41467-024-45235-w (PMC10844229; doi:10.1038/s41467-024-45235-w)
Supplement: Supplementary file 1 — Supplementary Information [file 41467_2024_45235_MOESM1_ESM.pdf]

Supplementary to 'Strong coupling between a microwave photon and a singlet-triplet qubit'

J. H. Ungerer,[1,2,3] A. Pally,[1,2] A. Kononov,[2] S. Lehmann,[4] J. Ridderbos,[2,5] P. P. Potts,[2,3]
C. Thelander,[4] K.A. Dick,[6] V.F. Maisi,[4] P. Scarlino,[7] A. Baumgartner,[2,3] and C. Schönenberger[2,3,8]
[1]*Equal contributions. Emails: jungerer@g.harvard.edu, alessia.pally@unibas.ch*
[2]*Department of Physics, University of Basel, Klingelbergstrasse 82 CH-4056, Switzerland*
[3]*Swiss Nanoscience Institute, University of Basel, Klingelbergstrasse 82 CH-4056, Switzerland*
[4]*Solid State Physics and NanoLund, Lund University, Box 118, S-22100 Lund, Sweden*
[5]*Current address: MESA+ Institute for Nanotechnology, University of Twente, P.O. Box 217, 7500 AE Enschede, The Netherlands*
[6]*Centre for Analysis and Synthesis, Lund University, Box 124, S-22100 Lund, Sweden*
[7]*Institute of Physics and Center for Quantum Science and Engineering, Ecole Polytechnique Fédérale de Lausanne, CH-1015 Lausanne, Switzerland*
[8]*nanoelectronics.unibas.ch*
(Dated: January 11, 2024)

I. ADDITIONAL FIGURES

Figure S1, Figure S2 and Figure S3 are beign refereed to in the methods section of the main text. They provide additional information accompanying the manuscript.

II. INAS CRYSTAL-PHASE NANOWIRES

InAs nanowires with controlled crystal structure were grown by metal-organic vapor phase epitaxy (MOVPE) from Au aerosol nanoparticles with a nominal diameter of 30 nm deposited on InAs 111B substrates. After annealing, nanowires were grown at 460 °C by introducing trimethylindium (TMIn) at a molar fraction of $\chi_{\mathrm{TMIn}} = 1.8 \cdot 10^{-6}$ and Arsine (AsH_3) at a molar fraction of $\chi_{\mathrm{AsH_3}} = 1.2 \cdot 10^{-4}$. Crystal-phase switching is realized by modifying the AsH_3 molar fractions from $\chi_{\mathrm{AsH_3}} = 2.5 \cdot 10^{-2}$ for zinc blende to $\chi_{\mathrm{AsH_3}} = 2.2 \cdot 10^{-5}$ for wurtzite, with 15 s waiting steps under AsH_3. The wurtzite barrier growth time is 54 s and the zinc blende segment growth time is 360 s. Deposited at the same growth temperature, the GaSb shell was grown for 40 minutes with respective molar fractions of trimethylgallium (TMGa) $\chi_{\mathrm{TMGa}} = 2.7 \cdot 10^{-6}$ and trimethylantimony (TMSb) $\chi_{\mathrm{TMSb}} = 3.1 \cdot 10^{-5}$. As the nanowires in this work were grown from randomly deposited Au seed particles, a variability in the local growth conditions was present that affect nanowire growth rate and segment lengths. However, by adding the GaSb shell that selectively deposits on zinc blende surfaces, it is possible to identify nanowires with desired segment lengths, and to accurately position contacts and local gates to these [1]. The GaSb-shell is then removed before contacting by a wet-etching process using MF-319 developer [1, 2].

By growing nanowires in arrays, such as from lithographically defined Au particles, the variability in seg-

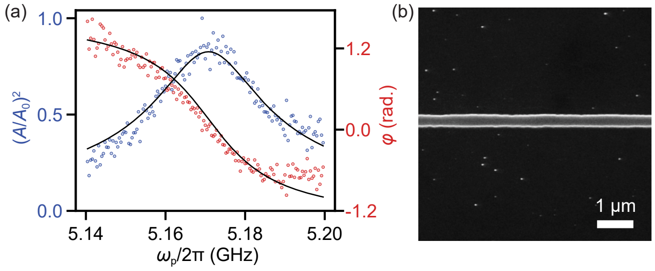

FIG. S1. **Resonator of device A.** (a) Resonance curve of the resonator in Coulomb blockade in amplitude A/A_0 (blue) and phase φ (red). The black lines are simultaneous fits to the data using input-ouput theory. (b) Scanning electron micrograph of the resonator center conductor.

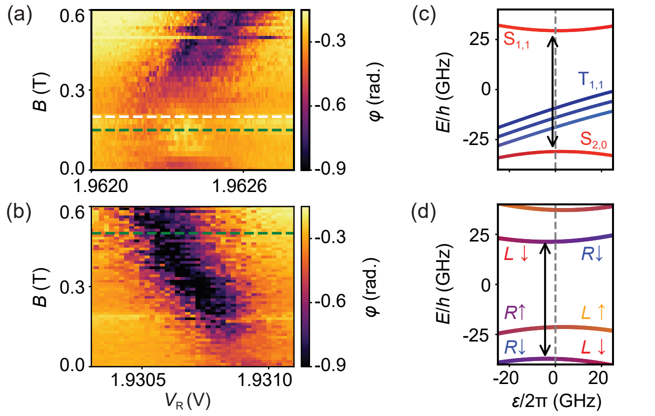

FIG. S2. **Dispersive read-out at low magnetic field.** Resonator phase in dependence of the right gate voltage V_R and magnetic field B for even (a) and odd (b) occupation of the DQD of device B. For the odd occupation the IDT shifts to lower V_R from $B = 0$. The IDT of the even occupation stays nearly independent of magnetic field until around 0.2 T (white dashed line), from where it starts moving to more positive V_R. Energy level diagram for the even (c) and odd (d) configuration at 0.15 T and 0.5 T (green dashed line). The arrow marks the transition the resonator is sensitive to, where the ground state energy level has maximum curvature.

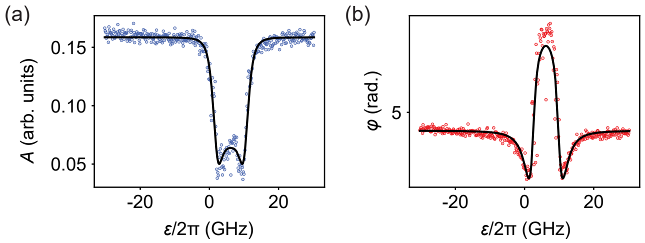

FIG. S3. **Fit of input-output theory to the data.** Input-output theory (solid lines) simoultaneously fitted to the resonator amplitude A (a) and the resonator phase φ (b) as a function of detuning ε of the even configuration at 0.25 T.

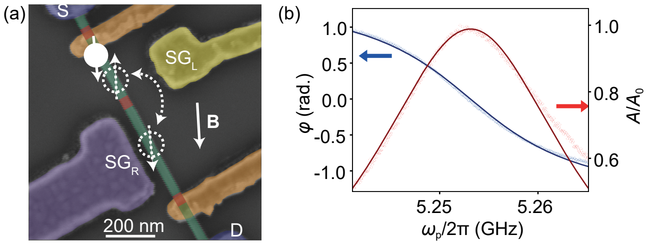

FIG. S4. **Device B** (a) False colored SEM-image of the device. The NW (green) is divided into two segments by an in-situ grown tunnel barrier (red), thus forming the DQD system. The NW ends are contacted by two Ti/Au contacts (S,D) and the NW segements can be electrically tuned by two Ti/Au sidegates SG_R (purple) and SG_L (yellow). A high-impedance, half-wave resonator is connected to SG_R. Top gates (orange) are kept at a constant voltage of -0.05 V. The magnetic field is applied in-plane at an angle of $\sim 60°$ to the NW. The arrows illustrate an even charge configuration with the two degenerate DQD states $T^+_{1,1}$ and $S_{2,0}$. (b) Resonance curve of the resonator in Coulomb blockade in amplitude A/A_0 (red) and phase φ (blue).

ment lengths can be greatly reduced [3]. The electron mobility in these nanowires is primarily limited by the wurtzite tunnel barriers and surface scattering. InAs nanowires with a pure zinc-blende crystal phase grown by a corresponding method show a room-temperature field-effect mobility of approximately 2000 cm^2/Vs [4].

In total, we have fabricated 4 nanowire devices coupled to a high-impedance resonator. All nanowires demonstrated well-defined double-quantum dots as expected from their barrier design. Out of these 4 devices, two were investigated at elevated magnetic-field strengths and both of them showed similar behavior as discussed in the manuscript.

III. HAMILTONIAN IN THE ODD CHARGE PARITY

In the main text, we elaborate on the Hamiltonian describing the double quantum dot (DQD) for an even charge occupation. This section provides the description for an *odd* number of electrons which is used in order to obtain Fig. S2(d). In this case, the total electron spin is 1/2 which can be modelled by one electron with a half spin. This electron can reside either on the left dot or on the right dot [5]. Therefore, a suitable basis is $\{|L\uparrow\rangle, |L\downarrow\rangle, |R\uparrow\rangle, |R\downarrow\rangle\}$, where L/R denotes whether the charge resides in the left dot or on the right dot, and $\uparrow/\downarrow$ denotes whether the spin is aligned parallel or anti-parallel with the magnetic field B.

The Hamiltonian describing the electron can be decomposed into three parts as

$$\mathcal{H}_{\text{odd}} = \mathcal{H}^0_{\text{odd}} + \mathcal{H}^Z_{\text{odd}} + \mathcal{H}^{\text{SO}}_{\text{odd}} \tag{1}$$

The first part of the Hamiltonian describes the spin-independent charge which can be written using the the charge Pauli matrices $\hat{\tau}_{x,y,z}$ as

$$\mathcal{H}^0_{\text{odd}} = \frac{\hbar\epsilon}{2}\hat{\tau}_z + \hbar t_c \hat{\tau}_x. \tag{2}$$

Here, the diagonal terms are proportional to the detuning $\hbar\epsilon = E_R - E_L$ which is the difference between the electrostatic potential of the electron residing in the right and left dot. The off-diagonal terms are given by $\hbar t_c$, which is the spin-conserving tunnel rate.

In the presence of a magnetic-field, $\mathcal{H}^Z_{\text{odd}}$ comes into effect. This term describes the Zeeman energy of the electron and is given by

$$\mathcal{H}^Z_{\text{odd}} = \frac{1}{2} g_{L,R}\mu_B B \hat{\sigma}_z, \tag{3}$$

where g_L and g_R are the site-dependent Landé g-factors, μ_B is the Bohr magneton and $\hat{\sigma}_{x,y,z}$ are the spin Pauli matrices. The Zeeman energy lifts the spin degeneracy and hence four spin-polarized levels are observed as shown in Fig. S2(d). As explained in the methods section, unequal g factors $g_L \neq g_R$ result in a shift of the avoided level crossings originating from spin-conserving tunneling. This results in a slope of the observed interdot transition as a function of gate voltage (detuning) and field from zero field onward.

IV. ANALYSIS OF DEVICE B

In this section we will discuss device B, which showed qualitatively similar behavior as device A which is discussed in the main text. Device B is shown in Fig. S4(a), including a false-colored SEM-image of the crystal-phase defined NW DQD. The DQD is hosted in the 280 nm and 380 nm long zincblende segments (green), separated by 30 nm long wurtzite (red) tunnel barriers. A high-impedance, half-wave coplanar-waveguide resonator is capacitively coupled to the DQD at its voltage anti-node via sidegate SG_R. One more side gate SG_L (yellow) allows to tune the electrostatic potential and there are two top gates (orange) kept at constant voltage of -0.05. We show the bare resonance curve in Fig. S4(b) and extract the bare resonance frequency $\omega_0/2\pi = 5.25308 \pm$

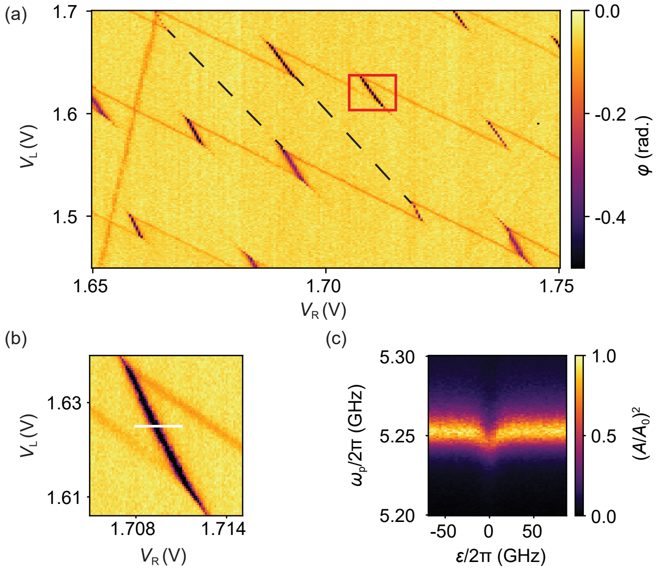

FIG. S5. **Dispersive sensing of the DQD at B = 0.** (a) Charge stability diagram of the device, in which the resonator phase φ is measured as a function of the SG voltages V_R and V_L. The negative slopes of the interdot transitions are due to the strong cross-capacitance of the larger gate SG_R. A zoom on the interdot transition pointed out by the red rectangle is shown in (b). (c) Resonator transmission $(A/A_0)^2$ versus probe frequency ω_p and detuning ϵ along the white line in (b). At the charge degeneracy point of the DQD, we observe a dispersive shift with respect to the bare resonance frequency.

0.00003 GHz and the bare decay rate $\kappa/2\pi = 23.2 \pm 0.8$ MHz. The main difference to device A is the weaker coupling of the resonator gate SG_R to the DQD and the smaller voltages applied to the top gates, which resulted in a stronger coupling of the DQD to the leads. Consequently, we observe a weaker spin-photon coupling and a larger qubit linewidth compared to device A. However, the behaviour of the singlet-triplet qubit in magnetic field is qualitatively the same, demonstrating that this kind of singlet-triplet is reproducible.

A. Charge-stability diagram

Fig. S5(a) shows the charge stability diagram of the DQD detected as a shift in the transmission phase φ of the resonator, plotted as a function of the two gate voltages V_L and V_R at a fixed probe frequency of 5.253 GHz, close to resonance. We observe a slanted honeycomb pattern, in which the inter-dot transition lines exhibit a negative slope due to the specific gate geometry (see Fig. S4(a)), which results in the right gate (V_R) coupling stronger to both dots than the left (V_L). Using a capacitance model [6, 7], we extract the gate-to-dot capacitances $C_{R2} = 2.5 \pm 0.4\,\mathrm{aF}$, $C_{L2} = 1.65 \pm 0.08\,\mathrm{aF}$, $C_{R1} = 10.1 \pm 0.6\,\mathrm{aF}$ and $C_{L1} = 2.0 \pm 0.2\,\mathrm{aF}$.

In Fig. S5(c) we show the resonator response while varying the probe frequency ω_p and changing the detuning ϵ along the white line in Fig. S5(b). By fitting input-output theory to this particular IDT, we extract the inter-dot tunnel coupling $t = 4.40 \pm 0.06$ GHz, charge-photon coupling $g_0 = 150 \pm 3$ MHz, and charge qubit linewidth $\gamma = 1.5 \pm 0.5$ GHz.

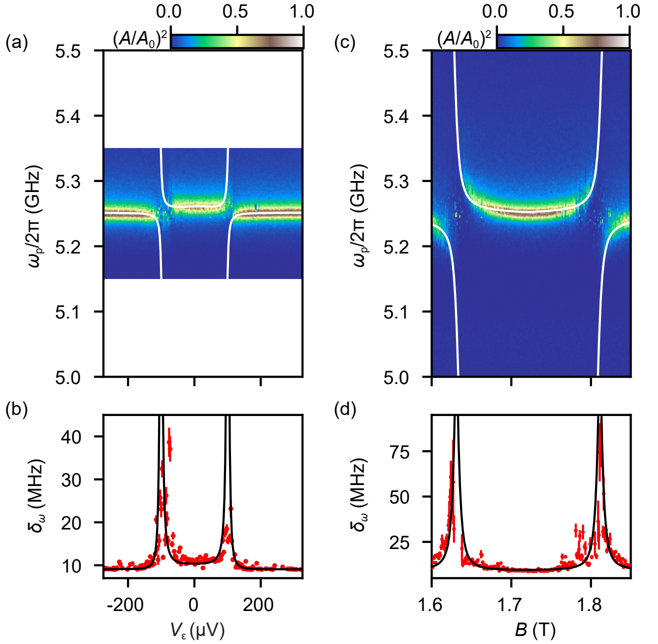

FIG. S6. **Avoided crossing as function of gate voltage and field.** (a) Resonator transmission at a fixed magnetic field $B = 1.67\,\mathrm{T}$ as function of detuning gate voltage V_ϵ. (b) Linewidths δ_ω extracted from (a). (c) Resonator transmission at a fixed detuning voltage corresponding to $\epsilon' = 1.65\,\mathrm{GHz}$ as function of magnetic field. (d) Linewidths δ_ω extracted from (c). Using a Jaynes-Cummings model fit, we extract the following parameters for (a) and (b): $g_0/2\pi = 164 \pm 6\,\mathrm{MHz}$, $t/2\pi = 1.34 \pm 0.05\,\mathrm{GHz}$, $\gamma = 317 \pm 28\,\mathrm{MHz}$, $\kappa = 18.2 \pm 0.2\,\mathrm{MHz}$. This results in a resonant coupling strength of $g(\omega_q = \omega_0) = 83 \pm 4\,\mathrm{MHz}$ when correcting for the mixing angle. From (c) and (d), we extract $g(\omega_q = \omega_0)/2\pi = 158 \pm 3\,\mathrm{MHz}$, $t(B = B_0)/2\pi \sim 0$, $\gamma/2\pi = 269 \pm 16\,\mathrm{MHz}$, $\kappa/2\pi = 18.6 \pm 0.2\,\mathrm{MHz}$. The larger value of the coupling strength in the magnetic-field (c,d) sweep compared to the detuning sweep (a,b) is attributed to the smaller mixing angle and reflected by the larger splitting at the anti-crossing in (c) compared to (a). Given the input power $P_\mathrm{in} = -133\,\mathrm{dBm}$, the average number of photons in these measurements is $n < 0.25$ (see methods of main part).

B. Avoided crossing

As illustrated in Fig. S7(c), the DQD can be operated as a singlet-triplet qubit when placed into a magnetic field. The qubit frequency $\omega_q = \Delta_{SO}/\hbar$ can be brought into resonance with the cavity frequency ω_0 at $B \approx 1.7$ T, as discussed in more detail below. At the reso-

nance condition ($\omega_q \sim \omega_0$), an anti-symmetric (bonding) and a symmetric (anti-bonding) qubit-photon superposition are formed. Consistently, at a field of $B \approx 1.7\,\mathrm{T}$, we observe an anti-crossing between the resonator and the singlet-triplet qubit. Figure S6(a) shows the anti-crossing as a function of the detuning voltage V_ϵ at constant magnetic field $B = 1.67\,\mathrm{T}$. By fitting a lorentzian to each trace of fixed detuning, we extract the resonance frequencies $\omega_{\Psi_\pm}$ and linewidths δ_ω (transmission and phase). Simultaneously, we fit the transition frequencies (dashed, white curves in Fig. S6(a)) and linewidths (solid, black curve in Fig. S6(b)) to the Jaynes-Cummings model. The transition frequencies are fitted as described in the methods section in the main text and linewidth of the transitions from the ground state to the predominantly photon-like dressed state $|\psi_-\rangle$ is given by

$$\begin{aligned}\delta_\omega &= |\langle\psi_-|g,1\rangle|^2\,\kappa + |\langle\psi_-|e,0\rangle|^2\,2\gamma \\ &= \cos^2(\theta)\,\kappa + \sin^2(\theta)\,2\gamma,\end{aligned} \tag{4}$$

where $\theta = \frac{1}{2}\tan^{-1}\left(\frac{2g}{\omega_q-\omega_0}\right)$ [8]. The fit parameters are given in the caption of Fig. S6.

In Fig. S6(c), we show the same anti-crossing as a function of B at a fixed detuning $\epsilon/2\pi \sim 1.65\,\mathrm{GHz}$. To extract the spin-photon coupling strength and qubit linewidth from this second measurement, we characterize the effective qubit transition frequency around the minimum $t_0 = t(B_0)$ by $\omega_q(B) = \sqrt{(2t_0)^2 + (\alpha_B(B-B_0))^2}$, where we introduce the heuristic scaling factor α_B . With this additional free parameter, we fit the Jaynes-Cummings model (dashed, white curves in Fig. 3(c) and solid, black curve in Fig. 3(d)) and extract the parameters described in the caption of Fig. S6.

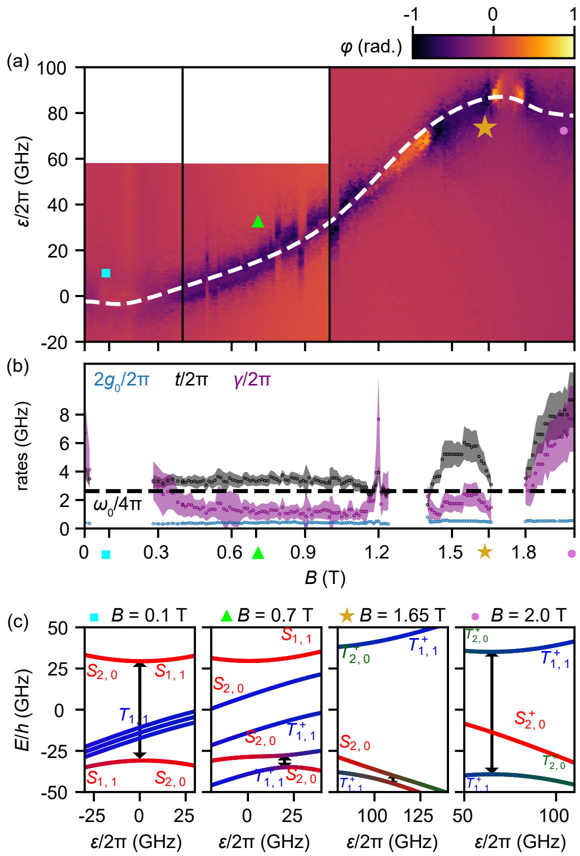

FIG. S7. **Magnetospectroscopy of the singlet-triplet qubit.** a) Resonator phase φ as a function of the magnetic field B and detuning ϵ. The white dashed line is a fit of the effective two-electron Hamiltonian to the data. b) Extracted tunnel rate $2t/2\pi$ (black), twice the qubit-photon coupling $2g_0/2\pi$ (cyan) and qubit linewidth $\gamma/2\pi$ (purple). Half the bare resonator frequency is indicated by the black dashed line. (c) Two-electron energy level diagrams at various magnetic fields with the corresponding field strength indicated in (a) and (b) by the given symbols. A constant offset of 20 GHz and 30 GHz was added to the energy levels at 1.65 T and 2.0 T, respectively.

C. Magnetospectroscopy

In this section we analyse the magnetospectroscopy of device B analogously to device A. We measure the resonator phase φ as a function of the detuning ϵ and the magnetic field, as plotted in Fig. S7(a). Resonator transmission and phase are simultaneously fitted to input-output theory and the qubit-photon coupling, qubit tunnel coupling and qubit linewidth are extracted. Descriptions to the method and formulas can be found in the main text and methods section.

We observe a qualitatively similar curve shape to device A in the main text. Again, we model the DQD by an effective two electron Hamiltonian which allows us to fit the gate voltage and field dependence of the IDT (white dashed line in Fig. S7(a)). We find that the magneto-dispersion of the IDT is well fitted using the following fit parameters namely the spin-conserving singlet and triplet tunnel rates $t_c^S/2\pi \approx 29\,\mathrm{GHz}$, and $t_c^T/2\pi \approx 37\,\mathrm{GHz}$, the singlet-triplet coupling rate $t_{\mathrm{SO}}/2\pi \approx 5\,\mathrm{GHz}$, the electron g-factors of the right and left dots, $g_R \approx 1.8$ and $g_L \approx 2.8$, as well as the singlet-triplet energy splitting $\Delta_{\mathrm{ST}}/2\pi \approx 79\,\mathrm{GHz}$. As for device A, these fit parameters are consistent with parameters obtained previously in this material system [9–13].

As described in the main text, we extract the the qubit tunnel amplitude t, the qubit linewidth γ, and the qubit-photon coupling strength g as a function of B, which we plot in Figure S7(b). A notable difference to device A, is the higher tunnel rate of device B. Unlike device A, device B has a qubit frequency predominantly above the resonator frequency and therefore anti-crosses only in small regions of the dispersion.

We will now shortly discuss the different regimes of the qubit, analogue to device A. Fig. S7(c) shows the corresponding DQD level structure based on the fit parameters as a function of ϵ for different magnetic field.

At a low magnetic fields around $B = 0.1\,\mathrm{T}$, we observe

again a singlet charge qubit with Zeeman-split triplets in the weak coupling limit. Again, we observe the characteristic double-dip structure between $B \sim 0.03\,\mathrm{T}$ and $B \sim 0.3\,\mathrm{T}$ of an even IDT.

As shown in the second panel of Fig. S7(c) at high enough field $T^+_{1,1}$ becomes the ground state for $\epsilon < 0$. The spin-orbit interaction couples the singlet and triplet states, leading to an anti-crossing between $S_{2,0}$ and $T^+_{1,1}$.

Consistent with the interpretation of the formation of a singlet-triplet qubit, we measure an approximately constant tunneling rate t between $B \sim 0.5\,\mathrm{T}$ and $B \sim 1.1\,\mathrm{T}$. We extract the average spin-orbit tunneling to be $\bar{t}_{\mathrm{so}} = 4.0 \pm 0.3\,\mathrm{GHz}$. At $B \approx 1.3\,\mathrm{T}$, χ becomes positive. This is interpreted as a drop of the tunnel rate below the resonator frequency, $2t < \omega_0$. This decline in t is not captured by our simplified Hamiltonian model and we speculate that changes in the orbital structure of a many-electron DQD could be the reason.

At a magnetic field of $B \approx 1.7\,\mathrm{T}$, we observe a resonant interaction between the resonator and the singlet-triplet qubit leading to the anti-crossing as discussed in section IV B. As seen in the level structure in Fig. S7(c) at $B = 1.65\,\mathrm{T}$, the triplet state $T^+_{2,0}$ becomes relevant. This results in a level repulsion between $T^+_{2,0}$ and $T^+_{1,1}$ and hence leads to a reduced splitting between the $S_{2,0}$ level and the $T^+_{1,1}$ states. In Fig. S7(c), this is illustrated by the smaller level gap (black arrow) compared to the one at $B = 0.7\,\mathrm{T}$.

The level structure at very large magnetic fields is plotted at $B \approx 2\,\mathrm{T}$ in the right panel of Fig. S7(c). Here, we observe the triplet charge qubit. As discussed in the main text, the triplet charge qubit has a larger frequency detuning from the resonator frequency than the singlet-triplet qubit, leading to a smaller resonator shift.

BIBLIOGRAPHY

[1] David Barker, Sebastian Lehmann, Luna Namazi, Malin Nilsson, Claes Thelander, Kimberly A Dick, and Ville F Maisi. Individually addressable double quantum dots formed with nanowire polytypes and identified by epitaxial markers. *Applied Physics Letters*, 114(18), 2019.

[2] C Gatzke, SJ Webb, K Fobelets, and RA Stradling. In situ raman spectroscopy of the selective etching of antimonides in gasb/alsb/inas heterostructures. *Semiconductor science and technology*, 13(4):399, 1998.

[3] Malin Nilsson, I-Ju Chen, Sebastian Lehmann, Vendula Maulerova, Kimberly A Dick, and Claes Thelander. Parallel-coupled quantum dots in inas nanowires. *Nano letters*, 17(12):7847–7852, 2017.

[4] Claes Thelander, Philippe Caroff, Sébastien Plissard, Anil W Dey, and Kimberly A Dick. Effects of crystal phase mixing on the electrical properties of inas nanowires. *Nano letters*, 11(6):2424–2429, 2011.

[5] W. G. van der Wiel, S. De Franceschi, J. M. Elzerman, T. Fujisawa, S. Tarucha, and L. P. Kouwenhoven. Electron transport through double quantum dots. *Reviews of Modern Physics*, 75(1):1–22, December 2002. doi: 10.1103/RevModPhys.75.1.

[6] Wilfred G Van der Wiel, Silvano De Franceschi, Jeroen M Elzerman, Toshimasa Fujisawa, Seigo Tarucha, and Leo P Kouwenhoven. Electron transport through double quantum dots. *Reviews of modern physics*, 75(1):1, 2002.

[7] Pasquale Scarlino, Jann H Ungerer, David J van Woerkom, Marco Mancini, Peter Stano, Clemens Müller, Andreas J Landig, Jonne V Koski, Christian Reichl, Werner Wegscheider, et al. In situ tuning of the electric-dipole strength of a double-dot charge qubit: Charge-noise protection and ultrastrong coupling. *Physical Review X*, 12(3):031004, 2022.

[8] Alexandre Blais, Ren-Shou Huang, Andreas Wallraff, Steven M Girvin, and R Jun Schoelkopf. Cavity quantum electrodynamics for superconducting electrical circuits: An architecture for quantum computation. *Physical Review A*, 69(6):062320, 2004.

[9] Carina Fasth, Andreas Fuhrer, Lars Samuelson, Vitaly N Golovach, and Daniel Loss. Direct measurement of the spin-orbit interaction in a two-electron inas nanowire quantum dot. *Physical review letters*, 98(26):266801, 2007.

[10] S Nadj-Perge, SM Frolov, JWW Van Tilburg, J Danon, Yu V Nazarov, R Algra, EPAM Bakkers, and LP Kouwenhoven. Disentangling the effects of spin-orbit and hyperfine interactions on spin blockade. *Physical Review B*, 81(20):201305, 2010.

[11] Malin Nilsson, Florinda Viñas Boström, Sebastian Lehmann, Kimberly A Dick, Martin Leijnse, and Claes Thelander. Tuning the two-electron hybridization and spin states in parallel-coupled inas quantum dots. *Physical Review Letters*, 121(15):156802, 2018.

[12] Mircea Trif, Vitaly N Golovach, and Daniel Loss. Spin dynamics in inas nanowire quantum dots coupled to a transmission line. *Physical Review B*, 77(4):045434, 2008.

[13] Christian Jünger, Raphaëlle Delagrange, Denis Chevallier, Sebastian Lehmann, Kimberly A Dick, Claes Thelander, Jelena Klinovaja, Daniel Loss, Andreas Baumgartner, and Christian Schönenberger. Magnetic-field-independent subgap states in hybrid rashba nanowires. *Physical Review Letters*, 125(1):017701, 2020.
